# Circ_WWC3 overexpression decelerates the progression of osteosarcoma by regulating miR-421/PDE7B axis

**DOI:** 10.1515/biol-2021-0021

**Published:** 2021-03-12

**Authors:** Sihai Liu, Jing Zhang, Ting Zheng, Xiongneng Mou, Wenwei Xin

**Affiliations:** Department of Orthopedics, Wuhan Third Hospital, Tongren Hospital of Wuhan University, Wuhan, Hubei, 430060, China; Taizhou Vocational and Technical College, Taizhou, Zhejiang, 318000, China; Department Emergency, Taizhou First People’s Hospital, No. 218 Hengjie Road, Huangyan District, Taizhou, Zhejiang, 318020, China

**Keywords:** OS, circ_WWC3, miR-421, PDE7B

## Abstract

**Background:**

Emerging evidence has shown that circular RNAs (circRNAs) are vital regulators in osteosarcoma (OS) progression. However, the effects of circ_WWC3 in OS have not been explored. In this research, the functions and mechanisms of circ_WWC3 in OS were investigated.

**Methods:**

Quantitative reverse trancription polymerase chain reaction (qRT-PCR) was adopted to determine the levels of circ_WWC3, WW and WWC3 mRNA, miR-421, and phosphodiesterase 7B (PDE7B) mRNA. RNase R assay was used to determine the characteristic of circ_WWC3. Colony formation assay and 3-(4,5-dimethyl-2-thiazolyl)-2,5-diphenyl-2-*H*-tetrazolium bromide (MTT) assay were applied for cell growth. Transwell assay was performed for cell migration and invasion. Flow cytometry analysis was utilized for cell apoptosis. Western blot assay was conducted for the levels of apoptosis-related proteins and PDE7B protein. Dual-luciferase reporter assay was carried out to analyze the targeting relationship between miR-421 and circ_WWC3 or PDE7B. The murine xenograft model was established to explore the effect of circ_WWC3 *in vivo*.

**Results:**

Compared to normal tissues and cells, circ_WWC3 and PDE7B were downregulated in OS tissues and cells. Overexpression of circ_WWC3 or PDE7B suppressed OS cell growth, migration, and invasion and promoted apoptosis *in vitro*. Regarding the mechanism analysis, circ_WWC3 positively modulated PDE7B expression by targeting miR-421. MiR-421 overexpression restored the impacts of circ_WWC3 on OS cell growth, metastasis, and apoptosis. Inhibition of miR-421 repressed the malignant behaviors of OS cells by targeting PDE7B. In addition, circ_WWC3 inhibited the tumorigenicity of OS *in vivo*.

**Conclusion:**

Circ_WWC3 overexpression slowed the development of OS by elevating PDE7B via sponging miR-421.

## 1 Introduction

Osteosarcoma (OS) is a common devastating malignancy in the skeletal system, mainly occurring in adolescents or children [1]. OS is featured by rapid development and high metastasis potentiality, resulting in poor prognosis and high mortality [2]. Presently, although remarkable progress has been made in OS therapy due to the widespread use of adjuvant chemotherapy, surgery, and radiotherapy, the overall survival rate of OS patients remains dismal [3,4,5]. Therefore, elucidating the pathogenesis of OS and discovering novel effective strategies for OS are of great significance.

Circular RNAs (circRNAs) are a class of noncoding RNAs (ncRNAs), which are featured by covalently closed continuous loops [6]. Currently, mounting evidence has shown that circRNAs play critical roles in the modulation of multiple cellular mechanisms through the competitive endogenous RNAs (ceRNAs) mechanism [7]. Up to date, diverse circRNAs have been verified to participate in the carcinogenesis of OS. For instance, circ_0008792 exerted the antitumor effect in OS through regulating miR-711/ZFP1 axis [8]. CircTADA2A facilitated the viability and metastasis of OS cells through sponging miR-203-3p and elevating CREB3 [9]. These studies demonstrated that circRNAs played different roles in the carcinogenesis of OS. As a member of circRNA, circ_WWC3 (also termed as circ_0001910) was found to be aberrantly downregulated in OS through analyzing GEO dataset GSE96964. Nonetheless, the exact biological roles of circ_WWC3 in OS remain unclear.

MicroRNAs (miRNAs) are small ncRNAs that can suppress gene expression after transcription via recognizing the 3′ untranslated region (3′UTR) of target mRNAs [10,11]. MiR-421 has been demonstrated to act as a tumor promoter in multiple human tumors, such as hepatocellular carcinoma [12], breast cancer [13], gallbladder cancer [14], and nasopharyngeal carcinoma [15]. In OS, miR-421 was identified to promote the malignant characteristics of OS cells by targeting LTBP2 [16] or MCPIP1 [17]. Phosphodiesterase 7B (PDE7B) is implicated in the prognosis and development of various diseases [18,19,20]. Moreover, Wang et al. declared that PDE7B level was declined in OS patients [21]. However, the functions of PDE7B in OS development and the relation between miR-421 and PDE7B remain unknown.

The purpose of this work was to determine the expression patterns of circ_WWC3, miR-421, and PDE7B in OS tissues and cells and explore their functional roles and relationships in regulating the malignant behaviors of OS cells.

## 2 Materials and methods

### 2.1 Tissues acquisition

Fifty OS tissue specimens and adjacent non-tumor tissues specimens were obtained from the patients at Wuhan Third Hospital, Tongren Hospital of Wuhan University. All samples were stored at −80°C prior to use. The clinicopathological characteristics of OS patients are shown in Table 1.

**Table 1:** Correlation between circ_WWC3, miR-421, or PDE7B expression and clinicopathologic features of OS patients

Parameters	Groups	Numbers (*n* = 50)	Relative expression					
			Circ_WWC3		miR-421		PDE7B	
Age	≥25	6	2.7	*P* < 0.05	2.99	*P* < 0.05	2.25	*P* > 0.05
	<25	44	2.25		3.45		1.89	
Sex	Male	23	2.26	*P >* 0.05	3.63	*P* < 0.05	1.5	*P* < 0.05
	Female	27	2.33		3.11		2.45	
Stage	I	13	3.34	*P* < 0.01(I–II)	2.35	*P* < 0.01(I–II)	2.35	*P* < 0.01(I–II)
	II	26	2.26	*P* < 0.01(II–III)	3.75	*P* > 0.05(II–III)	1.51	*P* < 0.01(II–III)
	III	11	1.51		3.57		2.43	
Metastasis	Yes	31	1.82	*P* < 0.01	3.83	*P* < 0.01	2.04	*P* < 0.05
	No	19	3.09		2.69		1.77	


**Informed consent:** Informed consent has been obtained from all individuals included in this study.
**Ethical approval:** The research related to human use has been complied with all the relevant national regulations, institutional policies, and in accordance with the tenets of the Helsinki Declaration and has been approved by the Ethics Committee of Wuhan Third Hospital, Tongren Hospital of Wuhan University.

### 2.2 Cell culture

Human fetal-osteoblast cell line (hFOB 1.19) and OS cell lines (MG63, HOS, U2OS, and SaOS-2) were purchased from the American Type Culture Collection (ATCC, Manassas, VA, USA). The cells were cultured in Dulbecco’s modified Eagle’s medium (DMEM; Gibco, Rockville, MD, USA) supplemented with 10% fetal bovine serum (FBS; Gibco) and 1% penicillin–streptomycin (Gibco) at 37°C in a humidified incubator containing 5% CO_2_.

### 2.3 Quantitative reverse transcription polymerase chain reaction (qRT-PCR)

Total RNA was separated from tissues and cells using TRIzol reagent (Beyotime, Shanghai, China). RNA concentration was measured on NanoDrop 2000c spectrophotometer (Thermo Fisher Scientific, Waltham, MA, USA). Then RNAs were transcribed into cDNAs using M-MLV Reverse Transcriptase Kit (Promega, Madison, WI, USA) or TaqMan MicroRNA Reverse Transcription Kit (Applied Biosystems, Foster City, CA, USA). Next, qRT-PCR was manipulated utilizing SYBR^®^ Green Master Mix (Takara, Dalian, China) and specific primers (Sangon, Shanghai, China) on a StepOne Plus real-time PCR system (Applied Biosystems). The expression was computed based on the 2^−ΔΔCt^ method. The synthesized primers were: circ_WWC3: (F: 5′-TCGGGGAGTTTGTCTTTGAT-3′ and R: 5′-GCCTCCACTGTTCTCTTGGA-3′); WWC3: (F: 5′-TTTGGAGCAAAAGCAGTCAA-3′ and R: 5′-ATGCCACAGATTTTGCACAC-3′); miR-421: (F: 5′-GTCGCGCGGGUUAAUGCCTC-3′ and R: 5′-GGACATUAGUUGUCUGUAAATAG-3′); PDE7B: (F: 5′-CTGTTAAGTAGGCGGAAGTCAA-3′ and R: 5′-CGATCAGAATGCCAAATGTGTT-3′); glyceraldehyde 3-phosphate dehydrogenase (GAPDH): (F: 5′-GGAGCGAGATCCCTCCAAAAT-3′ and R: 5′-GGCTGTTGTCATACTTCTCATGG-3′); U6: (F: 5′-CTCGCTTCGGCAGCACA-3′ and R: 5′-AACGCTTCACGAATTTGCGT-3′). GAPDH or U6 was used as the internal control.

### 2.4 RNase R digestion assay

A total of 5 μg total RNA was treated with or without RNase R (2 U/μg; Epicentre, Madison, Wisconsin, USA) for 30 min at 37°C. Thereafter, qRT-PCR was implemented to detect the levels of circ_WWC3 and WWC3 mRNA.

### 2.5 Cell transfection

The overexpression plasmid of circ_WWC3 (circ_WWC3) and matched control Vector, the overexpression plasmid of PDE7B (PDE7B) and its control pcDNA, miR-421 mimics (miR-421) and miR-NC, miR-421 inhibitors (anti-miR-421) and anti-miR-NC, small interfering RNA targeting PDE7B (si-PDE7B), and si-NC were synthesized by Sangon. Lipofectamine 3000 (Invitrogen, Carlsbad, CA, USA) was used for the transfection of OS cells in reference to the guidelines of manufacturers.

### 2.6 Colony formation assay

Following the transfected OS cells (800 cells) being planted in a 6-well plate and cultivated in culture medium for about 2 weeks at 37°C, the emerged colonies were fixed in 4% paraformaldehyde (Sangon), stained with crystal violet (Sangon), and then photographed. The colonies were counted when more than 50 cells were seen.

### 2.7 3-(4,5-Dimethyl-2-thiazolyl)-2,5-diphenyl-2-*H*-tetrazolium bromide (MTT) assay

OS cells (5 × 10^3^ cells/well) transfected with indicated oligonucleotides and plasmids were sowed into 96-well plates and cultivated for 24 h. Then at 1-3 days, 20 µL MTT solution (Beyotime) was added to each well and cultured for another 4 h. Next, cell supernatant was removed and 150 µL DMSO (Sangon) was added to dissolve the formazan crystals. Lastly, the absorbance at 490 nm was detected with a microplate reader (Potenov, Beijing, China).

### 2.8 Transwell assay

About 1 × 10^4^ OS cells suspended in serum-free medium were added into the top compartment of transwell inserts (BD Bioscience, San Jose, CA, USA) pre-coated with (for invasion assay) or without (for migration assay) Matrigel (BD Biosciences). 600 μL culture medium was added into the bottom compartment as an attractive substance for cells in the top compartment. After 24 h, the cells migrated or invaded to the bottom surface were fixed in 4% paraformaldehyde (Sangon), dyed with crystal violet (Sangon), and then counted under a microscope at the magnification of 100×.

### 2.9 Flow cytometry analysis

The transfected MG63 and SaOS-2 cells were inoculated into 6-well plates and rinsed with cold PBS (Sangon). Then cells were resuspended and stained utilizing Annexin V-fluorescein isothiocyanate (FITC) Apoptosis Detection Kit (Vazyme, Nanjing, China) in line with the protocols of manufacturers. The apoptotic cells were determined with a flow cytometry (Beckman Coulter, Atlanta, GA, USA).

### 2.10 Western blot assay

Protein extraction was finished using RIPA buffer (Beyotime) and protein concentration determination was executed utilizing a BCA Protein Quantification Kit (Vazyme). 20 μg proteins were separated by 10% sodium dodecyl sulfonate-polyacrylamide gel (Solarbio, Beijing, China) electrophoresis. Next, the proteins were blotted onto polyvinylidene difluoride membranes (Pall Corporation, New York, NYC, USA). Afterward, the membranes were blocked utilizing 5% nonfat milk for 1 h at room temperature and incubated with primary antibodies against GAPDH (bs-2188R; 1:5,000; Bioss, Beijing, China), B-cell lymphoma-2 (Bcl-2; 1:2,000; bs-34012R; Bioss), BCL2-Associated X (Bax; 1:2,000; bs-0127R; Bioss), cleaved caspase-3 (bs-0081R; 1:2,000; Bioss), and PDE7B (bs-11576R; 1:2,000; Bioss) overnight at 4°C and secondary antibody (bs-0295M-HRP; 1:5,000; Bioss) for 1 h at room temperature, followed by ECL detection.

### 2.11 Dual-luciferase reporter assay

The binding sites between miR-421 and circ_WWC3 or PDE7B were predicted by online tools circinteractome and Starbase3.0. The sequences of circ_WWC3 and PDE7B including the wild-type (wt) or mutant (mut) binding sites of miR-421 were inserted into pmirGLO plasmid (Promega) to generate circ_WWC3-wt, PDE7B-wt, circ_WWC3-mut, and PDE7B-mut. Then the generated plasmids were transfected into MG63 and SaOS-2 cells together with miR-421 or miR-NC. After 48 h, the firefly and renilla luciferase activities were measured by Dual-Luciferase Reporter Assay Kit (Promega).

### 2.12 Murine xenograft model

The BALB/c nude mice (4–6 weeks old) were bought from the Laboratory Animal Resources, Chinese Academy of Sciences (Beijing, China), and assigned to 2 groups (*n* = 7). Then MG63 cells overexpressing circ_WWC3 were subcutaneously implanted into the mice. The volume of xenograft tumor was monitored every week and computed with the formula: (length × width^2^)/2. After 4 weeks, the mice were sacrificed and the weight of neoplasms was examined. The tissue samples were collected and preserved at −80°C.


**Ethical approval:** The research related to animal use has been complied with all the relevant national regulations and institutional policies for the care and use of animals and has been approved by the Ethics Committee of Animal Research of Wuhan Third Hospital, Tongren Hospital of Wuhan University.

### 2.13 Statistical analysis

Each experiment was conducted in triplicates. Data analysis was executed using GraphPad Prism 7 and the results were exhibited as mean ± standard deviation. The differences between two groups were estimated by Student’s *t*-test, while those among three groups were estimated using one-way analysis of variance followed by Tukey’s test. The linear correlations among the expression of circ_WWC3, miR-421, and PDE7B in OS tissues were evaluated by Spearman’s correlation coefficient analysis. Difference was considered as significant when *P* < 0.05.

## 3 Results

### 3.1 Circ_WWC3 has low expression in OS tissues and cells

**Figure 1:**
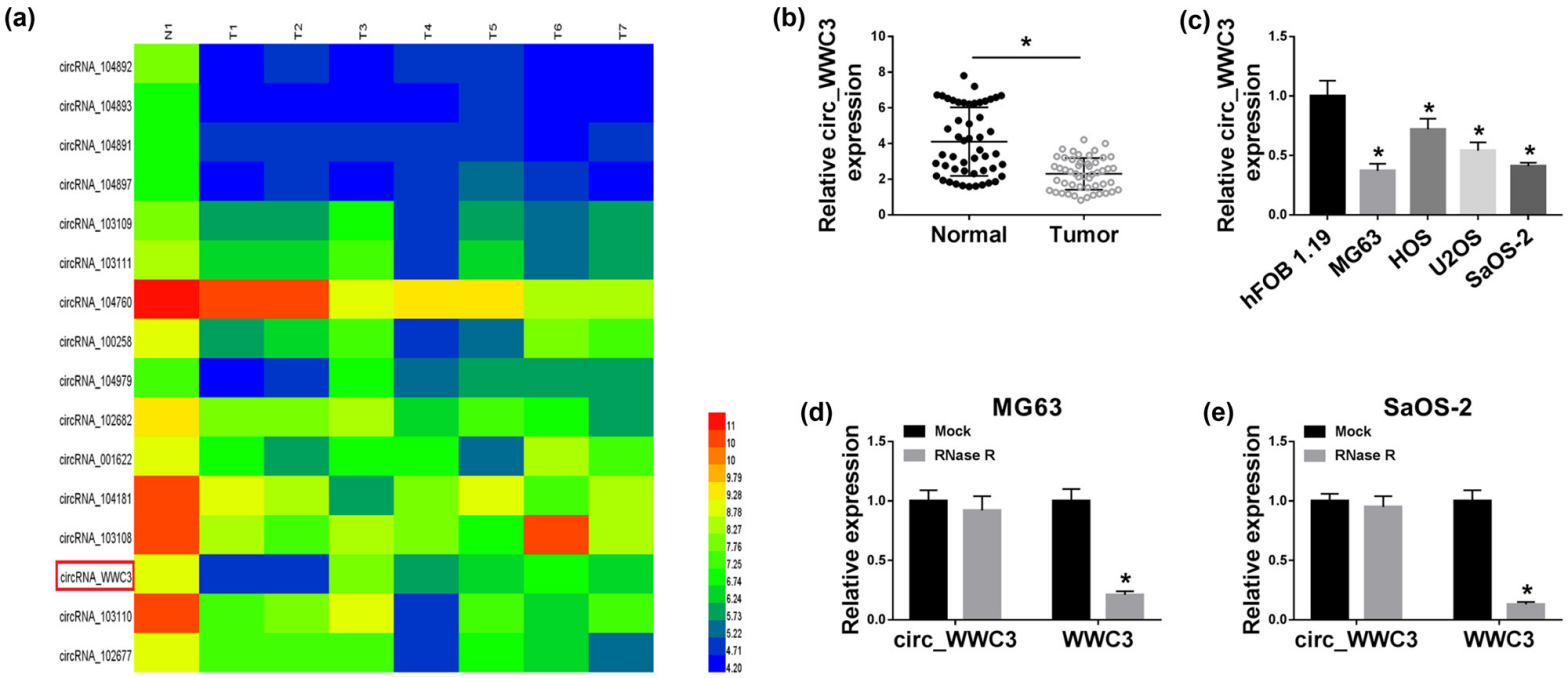
Circ_WWC3 was downregulated in OS tissues and cells. (a) GEO dataset GSE96964 exhibited that circ_WWC3 was downregulated in OS tissues. (b and c) qRT-PCR assay was conducted for the expression level of circ_WWC3 in OS tissues and cells, versus matched normal tissues and cells. (d and e) The levels of circ_WWC3 and WWC3 mRNA were determined using qRT-PCR after treatment with or without RNase R in MG63 and SaOS-2 cells. *P < 0.05.

Through analyzing GEO dataset GSE96964, we found that circ_WWC3 was downregulated in OS tissue samples (Figure 1a). Then the expression of circ_WWC3 in 50 OS tissues and adjacent normal tissues was measured by qRT-PCR, showing that circ_WWC3 was notably declined in OS tissues compared to normal tissues (Figure 1b). Moreover, we observed that circ_WWC3 was markedly decreased in OS cell lines (MG63, HOS, U2OS, and SaOS-2) compared to that in hFOB 1.19 cell line (Figure 1c). Given that circ_WWC3 expression in MG63 and SaOS-2 cells was lower than in HOS and U2OS cells, MG63 and SaOS-2 cells were selected for the subsequent experiments. Next, the feature of circ_WWC3 was analyzed by RNase R digestion assay. The data showed that circ_WWC3 was resistant to RNase R treatment, while WWC3 mRNA was evidently reduced after the treatment of RNase R in both MG63 and SaOS-2 cells (Figure 1d and e). All these results suggested that circ_WWC3 was stable, and the dysregulation of circ_WWC3 might be associated with OS progression.

### 3.2 Circ_WWC3 overexpression inhibited the malignant behaviors of OS cells

**Figure 2:**
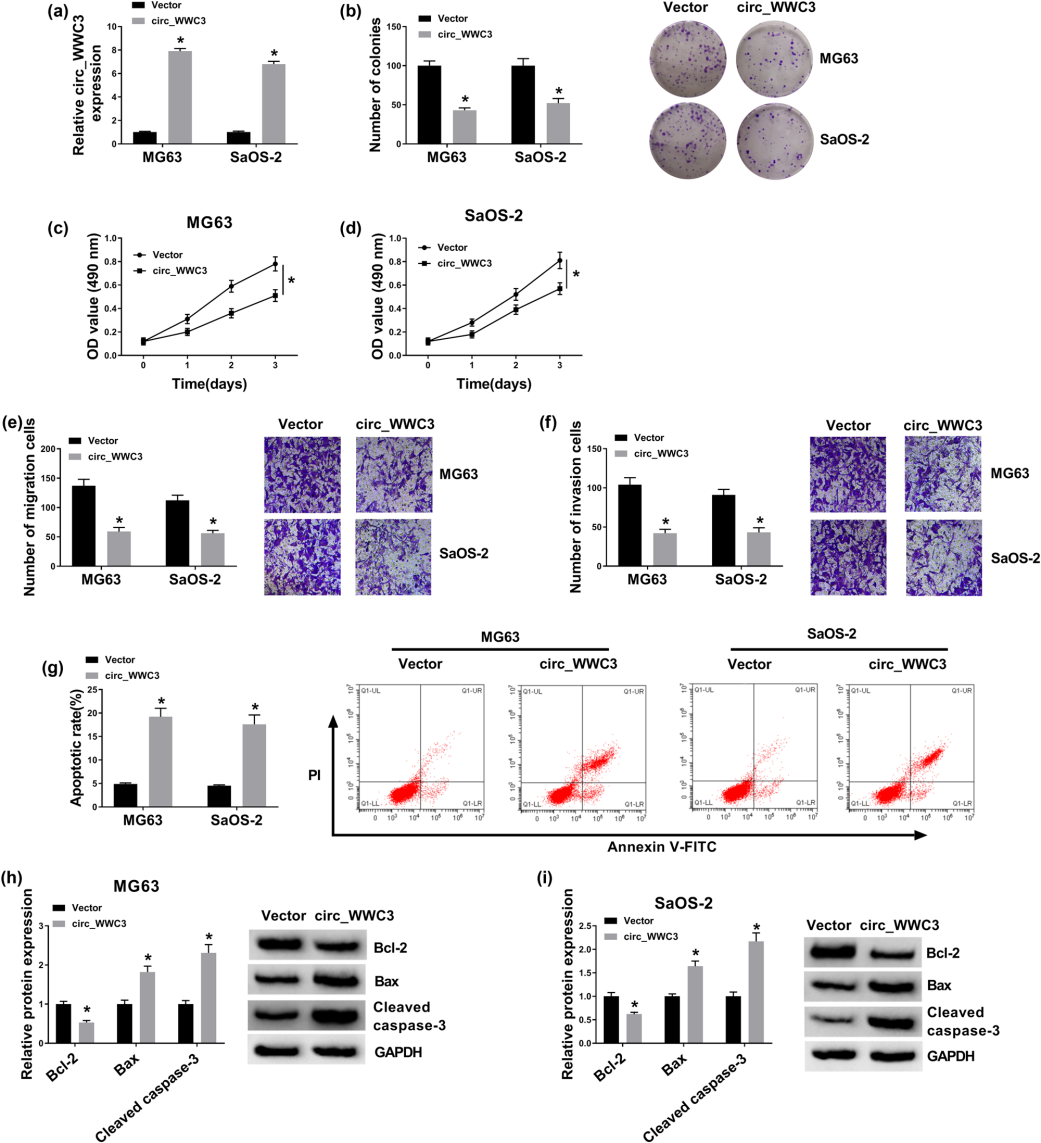
Circ_WWC3 overexpression suppressed cell colony formation, proliferation, migration, and invasion and promoted apoptosis in OS cells. Circ_WWC3 or Vector was transfected into MG63 and SaOS-2 cells. (a) Circ_WWC3 expression in MG63 and SaOS-2 cells was determined by qRT-PCR assay. (b) Cell colony formation, (c and d) proliferation, (e and f) migration, invasion, and (g) apoptosis in MG63 and SaOS-2 cells were evaluated by colony formation assay, MTT assay, transwell assay, and flow cytometry analysis, respectively. (h and i) The protein levels of Bcl-2, Bax, and cleaved caspase-3 in MG63 and SaOS-2 cells were measured via western blot assay. *P < 0.05.

To validate the precise biological functions of circ_WWC3 in OS cell progression, the overexpression vector of circ_WWC3 was transfected into MG63 and SaOS-2 cells. The transfection efficiency of circ_WWC3 was assessed through qRT-PCR analysis. The results exhibited that circ_WWC3 was conspicuously elevated in MG63 and SaOS-2 cells after circ_WWC3 transfection, indicating that the transfection was successful (Figure 2a). Colony formation assay showed that circ_WWC3 overexpression drastically repressed the colony formation capacity of MG63 and SaOS-2 cells compared to control groups (Figure 2b). MTT assay indicated that the proliferation of MG63 and SaOS-2 cells was markedly inhibited by the elevation of circ_WWC3 relative to control groups (Figure 2c and d). As demonstrated by transwell assay, the migration and invasion of MG63 and SaOS-2 cells were distinctly suppressed by upregulating circ_WWC3 in comparison with vector groups (Figure 2e and f). Flow cytometry analysis exhibited that circ_WWC3 overexpression obviously promoted the apoptosis of MG63 and SaOS-2 cells compared to control groups (Figure 2g). Moreover, we determined the levels of apoptosis-related proteins (including Bcl-2, Bax, and cleaved caspase-3) via western blot assay. The results displayed that circ_WWC3 overexpression decreased the expression of Bcl-2 and increased the expression of Bax and cleaved caspase-3 in both MG63 and SaOS-2 cells (Figure 2h and i). Collectively, circ_WWC3 overexpression inhibited OS cell colony formation, proliferation, migration and invasion, and induced apoptosis.

### 3.3 PDE7B overexpression relieved the malignant phenotypes of OS cells

**Figure 3:**
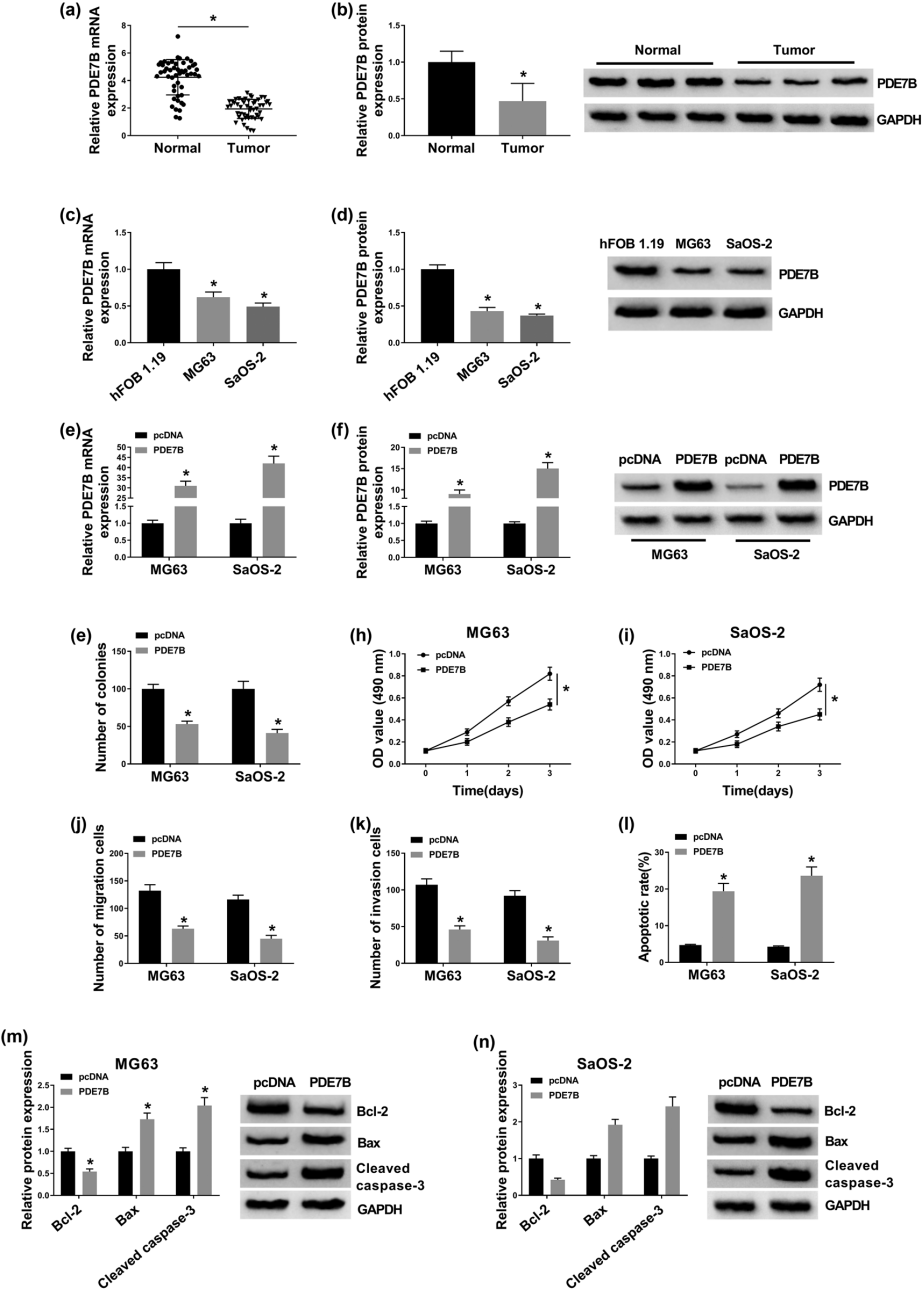
PDE7B overexpression suppressed OS cell colony formation, proliferation, migration, and invasion and promoted apoptosis. (a–d) The mRNA and protein levels of PDE7B in OS tissues, cells, and corresponding normal tissues and cells were measured by qRT-PCR assay and western blot assay, respectively. (e–n) MG63 and SaOS-2 cells were transfected with PDE7B or pcDNA. (e and f) The mRNA and protein levels of PDE7B in MG63 and SaOS-2 cells were detected via qRT-PCR assay or western blot assay. (g) Cell colony formation, (h and i) proliferation, (j and k) migration, invasion, and (l) apoptosis in MG63 and SaOS-2 cells were assessed through colony formation assay, MTT assay, transwell assay, and flow cytometry analysis, respectively. (m and n) Western blot analysis was performed for the levels of Bcl-2, Bax, and cleaved caspase-3 in MG63 and SaOS-2 cells. *P < 0.05.

Subsequently, we measured the mRNA and protein levels of PDE7B in OS tissues and adjacent normal tissues. The results of qRT-PCR analysis and western blot assay showed that PDE7B mRNA and protein levels were reduced in tumor tissues compared to normal tissues (Figure 3a and b). Likewise, the mRNA and protein levels of PDE7B were obviously reduced in MG63 and SaOS-2 cells compared to hFOB 1.19 cells (Figure 3c and d). Then PDE7B or pcDNA was successfully transfected into MG63 and SaOS-2 cells to elevate the expression of PDE7B. As presented in Figure 3e and f, PDE7B transfection evidently enhanced the mRNA and protein levels of PDE7B in MG63 and SaOS-2 cells. As illustrated by colony formation assay, MTT assay, and transwell assay, the abilities of colony formation, proliferation, migration, and invasion in MG63 and SaOS-2 cells were drastically suppressed by the overexpression of PDE7B (Figure 3g–k). Moreover, PDE7B elevation markedly induced the apoptosis of MG63 and SaOS-2 cells, concomitant with downregulation of antiapoptotic protein Bcl-2 and upregulation of pro-apoptotic proteins Bax and cleaved caspase-3 (Figure 3l–n). To sum up, PDE7B overexpression restrained OS cell growth and metastasis and facilitated apoptosis.

### 3.4 Circ_WWC3 positively regulated PDE7B expression by sponging miR-421 in OS cells

**Figure 4:**
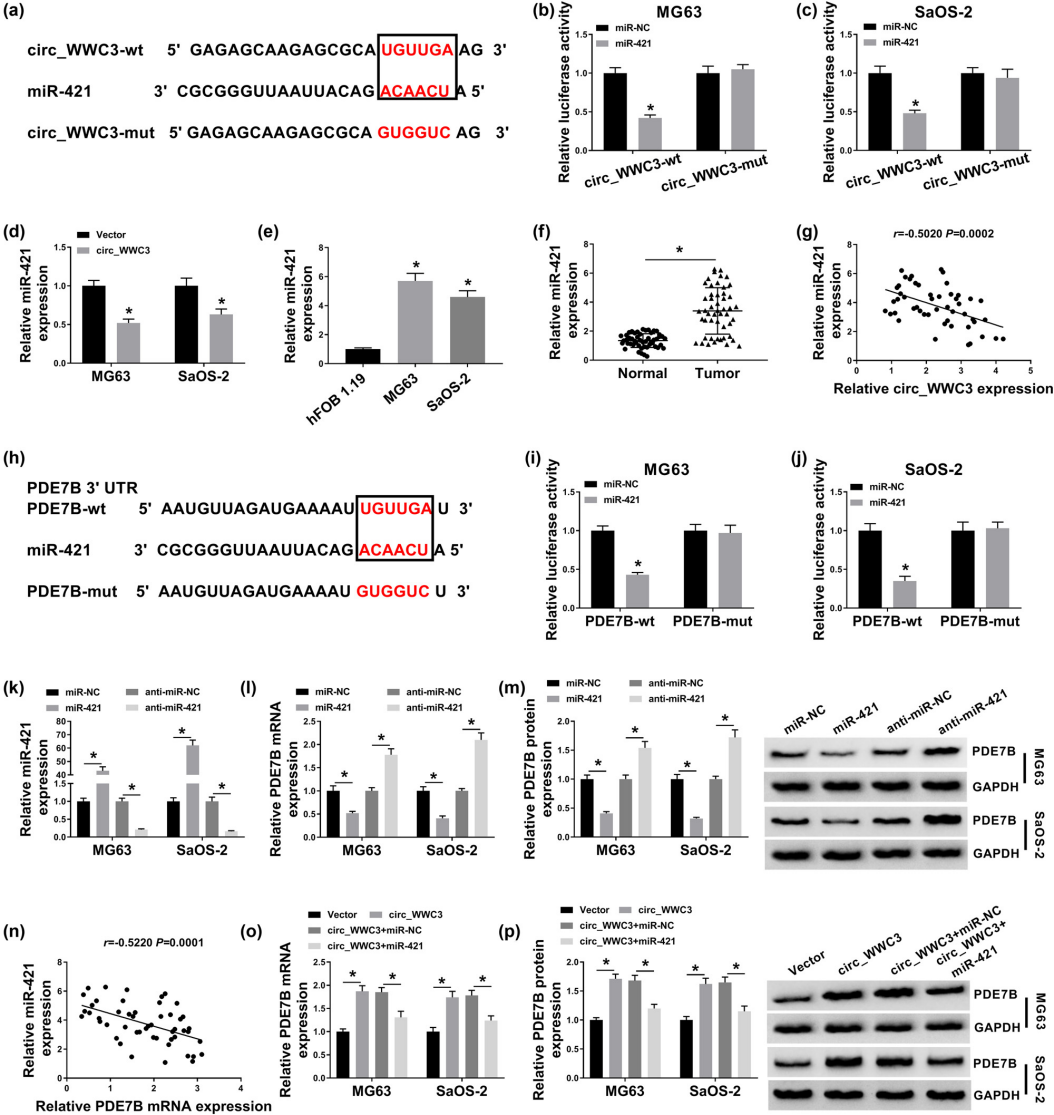
Circ_WWC3 facilitated PDE7B expression by interacting with miR-421 in OS cells. (a) The potential binding sites between circ_WWC3 and miR-421 were presented. (b and c) The luciferase activity in MG63 and SaOS-2 cells co-transfected with miR-421/miR-NC and circ_WWC3-wt/circ_WWC3-mut was measured through dual-luciferase reporter assay. (d) The expression of miR-421 in MG63 and SaOS-2 cells transfected with circ_WWC3 or Vector was determined by qRT-PCR assay. (e and f) The expression of miR-421 in OS cells, tissues, and corresponding normal cells and tissues was detected via qRT-PCR assay. (g) The correlation between the levels of miR-421 and circ_WWC3 in OS tissues was analyzed by spearman’s correlation coefficient analysis. (h) The complementary sequences between PDE7B and miR-421 were shown. (i and j) The luciferase activity in MG63 and SaOS-2 cells co-transfected with miR-421/miR-NC and PDE7B-wt/PDE7B-mut was measured using dual-luciferase reporter assay. (k–m) The levels of miR-421, PDE7B mRNA, and PDE7B protein in MG63 and SaOS-2 cells were measured by qRT-PCR assay or western blot assay. (n) The linear correlation between the levels of miR-421 and PDE7B in OS tissues was estimated by spearman’s correlation coefficient analysis. (o and p) Circ_WWC3, Vector, circ_WWC3 + miR-421, or circ_WWC3 + miR-NC was transfected into MG63 and SaOS-2 cells, and then the mRNA and protein levels of PDE7B were measured by qRT-PCR assay and western blot assay, respectively. *P < 0.05.

To further investigate the underlying mechanism of circ_WWC3 in the regulation of OS cell progression, bioinformatics software circinteractome (https://circinteractome.nia.nih.gov/) was used to analyze the potential target miRNAs of circ_WWC3. As displayed in Figure 4a, miR-421 contained the complementary sequences of circ_WWC3. Dual-luciferase reporter assay exhibited that miR-421 transfection obviously inhibited the luciferase activity of circ_WWC3-wt in MG63 and SaOS-2 cells, but did not affect the luciferase activity of circ_WWC3-mut, further confirming the combination between circ_WWC3 and miR-421 (Figure 4b and c). The overexpression of circ_WWC3 led to a marked suppression in miR-421 expression in MG63 and SaOS-2 cells compared to control groups (Figure 4d). Then we determined the expression of miR-421 in OS cells and tissues by qRT-PCR assay, exhibiting that miR-421 was notably elevated in OS cells and tissues compared to normal cells and tissues (Figure 4e and f). As estimated by Spearman’s correlation coefficient analysis, there was an inverse correlation between the levels of miR-421 and circ_WWC3 in OS tissues (Figure 4g). Through analyzing bioinformatics software Starbase3.0, we found PDE7B was a direct target gene of miR-421 and their potential binding sites were shown in Figure 4h. Dual-luciferase reporter assay was then conducted to verify this prediction. The results showed that the luciferase activity in MG63 and SaOS-2 cells was evidently decreased following the co-transfection of miR-421 and PDE7B-wt compared to miR-NC and PDE7B-wt co-transfected groups, whereas no change was observed in PDE7B-mut groups (Figure 4i and j). Then miR-421 or anti-miR-421 was successfully transfected into MG63 and SaOS-2 cells and the transfection efficiency was detected by qRT-PCR assay (Figure 4k). We observed that miR-421 overexpression strikingly decreased the mRNA and protein levels of PDE7B, while miR-421 inhibition exhibited the opposite results (Figure 4l and m). Spearman’s correlation coefficient analysis showed that miR-421 expression was negatively correlated with PDE7B mRNA expression in OS tissues (Figure 4n). Afterward, we transfected circ_WWC3, Vector, circ_WWC3 + miR-NC, or circ_WWC3 + miR-421 into MG63 and SaOS-2 cells to further analyze the relationships among circ_WWC3, miR-421, and PDE7B. As presented in Figure 4o and p, circ_WWC3 overexpression apparently elevated the mRNA and protein levels of PDE7B in MG63 and SaOS-2 cells, while the impacts were restored by upregulating miR-421. Taken together, circ_WWC3 promoted PDE7B expression via targeting miR-421 in OS cells.

### 3.5 MiR-421 overexpression rescued the inhibitory effect of circ_WWC3 on OS cell progression

**Figure 5:**
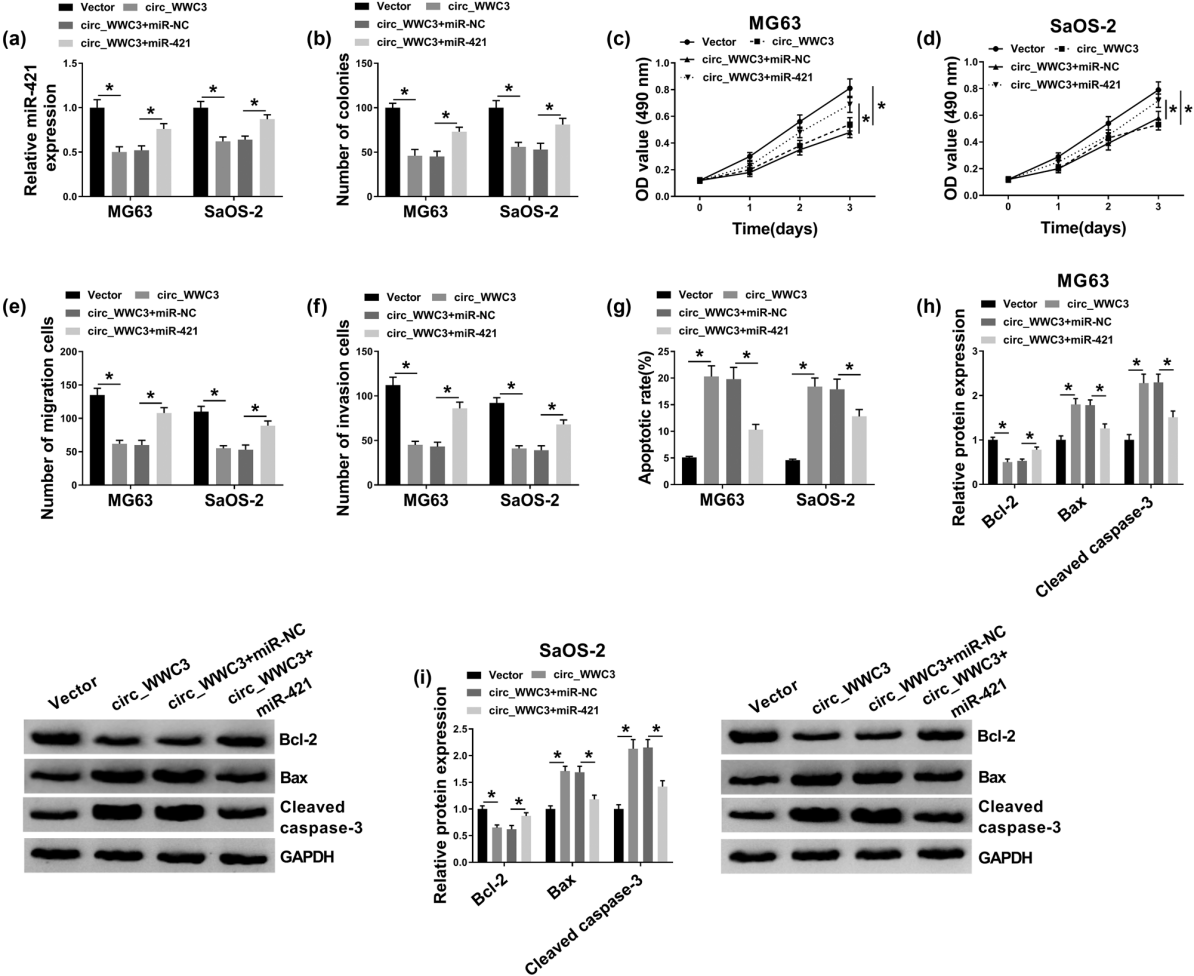
Circ_WWC3 overexpression repressed the malignant biological behaviors of OS cells by targeting miR-421. MG63 and SaOS-2 cells were transfected with Vector, circ_WWC3, circ_WWC3 + miR-NC, or circ_WWC3 + miR-421. (a) QRT-PCR analysis was conducted for the expression of miR-421 in MG63 and SaOS-2 cells. (b–g) The colony formation, proliferation, migration, invasion, and apoptosis of MG63 and SaOS-2 cells were detected using colony formation assay, MTT assay, transwell assay, and flow cytometry analysis, respectively. (h and i) Western blot analysis was performed for the protein levels of Bcl-2, Bax, and cleaved caspase-3 in MG63 and SaOS-2 cells. *P < 0.05.

To elucidate whether circ_WWC3 could inhibit OS cell progression by targeting miR-421, MG63 and SaOS-2 cells were divided into 4 groups: Vector, circ_WWC3, circ_WWC3 + miR-NC, and circ_WWC3 + miR-421. As demonstrated by qRT-PCR assay, circ_WWC3 overexpression markedly reduced miR-421 expression in MG63 and SaOS-2 cells, while miR-421 transfection restored the impact (Figure 5a). As illustrated by colony formation assay, MTT assay, transwell assay, and flow cytometry analysis, the suppressive roles of circ_WWC3 overexpression in cell colony formation, proliferation, migration, and invasion and the promotional role in apoptosis in MG63 and SaOS-2 cells were effectively overturned by upregulating miR-421 (Figure 5b–g). The results of western blot assay showed that the effects of circ_WWC3 overexpression on the protein levels of Bcl-2, Bax, and cleaved caspase-3 in MG63 and SaOS-2 cells were also restored by the elevation of miR-421 (Figure 5h and i). These results suggested that circ_WWC3 overexpression relieved the progression of OS cells by sponging miR-421.

### 3.6 MiR-421 inhibition suppressed OS cell progression by targeting PDE7B

**Figure 6:**
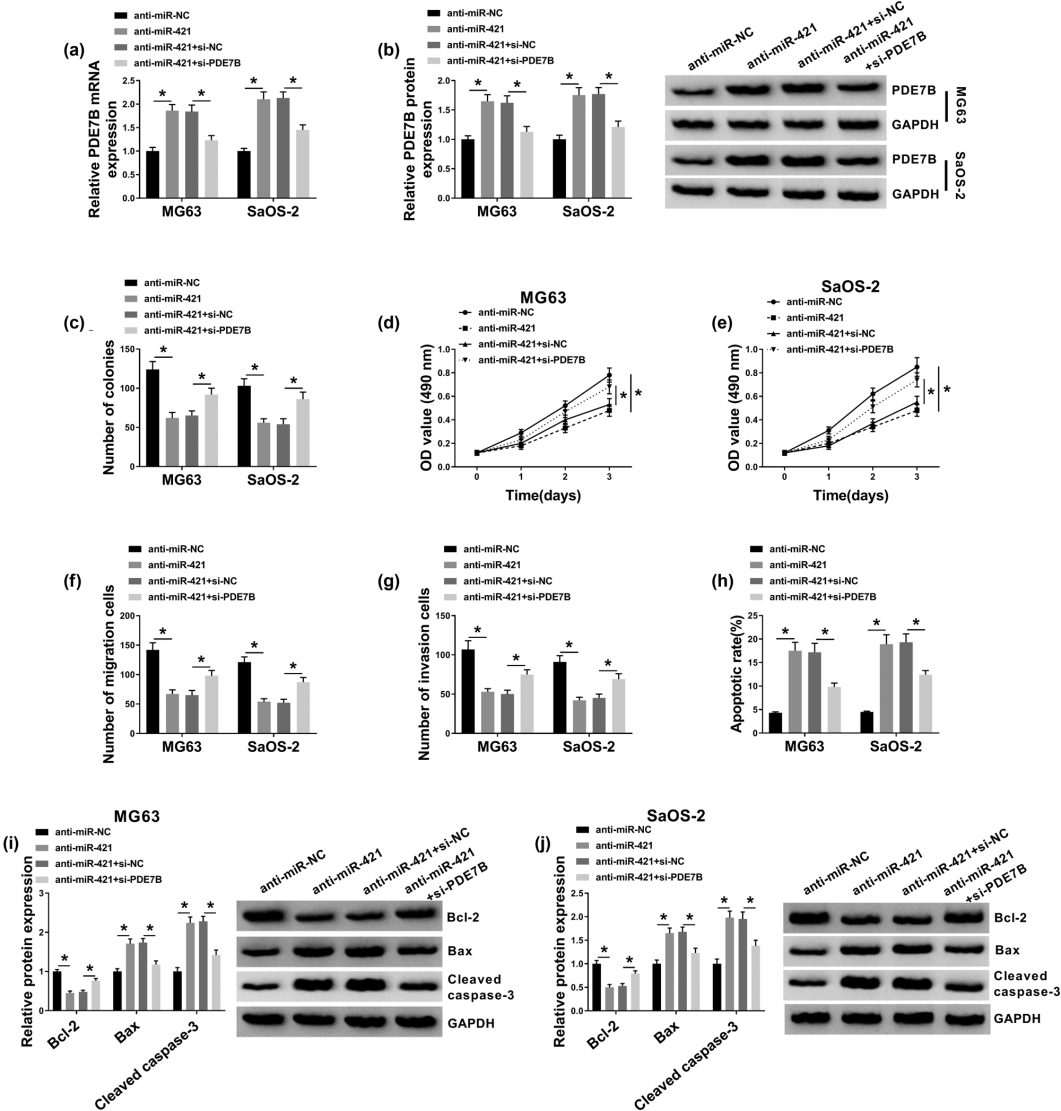
Inhibition of miR-421 suppressed the malignant phenotypes of OS cells by binding to PDE7B. MG63 and SaOS-2 cells were transfected with anti-miR-421, anti-miR-NC, anti-miR-421 + si-NC, or anti-miR-421 + si-PDE7B. (a and b) The mRNA and protein levels of PDE7B in MG63 and SaOS-2 cells were detected by qRT-PCR assay and western blot assay, respectively. (c–h) Cell colony formation, proliferation, migration, invasion, and apoptosis in MG63 and SaOS-2 cells were evaluated by colony formation assay, MTT assay, transwell assay, and flow cytometry analysis, respectively. (i and j) The protein levels of Bcl-2, Bax, and cleaved caspase-3 in MG63 and SaOS-2 cells were measured by western blot assay. *P < 0.05.

To further explore the relationship between miR-421 and PDE7B in OS cell progression, MG63 and SaOS-2 cells were treated with anti-miR-421, anti-miR-NC, anti-miR-421 + si-NC, or anti-miR-421 + si-PDE7B. As we observed in Figure 6a and B, the elevated mRNA and protein expression levels of PDE7B mediated by anti-miR-421 in MG63 and SaOS-2 cells were restored by si-PDE7B transfection. As demonstrated by colony formation assay, MTT assay, transwell assay, and flow cytometry analysis, miR-421 inhibition repressed cell colony formation, proliferation, migration, and invasion and induced apoptosis in MG63 and SaOS-2 cells, whereas the effects were abated by silencing PDE7B (Figure 6c–h). In addition, we found that miR-421 inhibition resulted in a remarkable reduction in Bcl-2 expression and a noteworthy elevation in Bax and cleaved caspase-3 expression in both MG63 and SaOS-2 cells, while the deficiency of PDE7B partially abrogated the effects (Figure 6i and j). Collectively, miR-421 inhibition decelerated the progression of OS cells by targeting PDE7B.

### 3.7 Circ_WWC3 overexpression blocked tumor growth of OS *in vivo*

**Figure 7:**
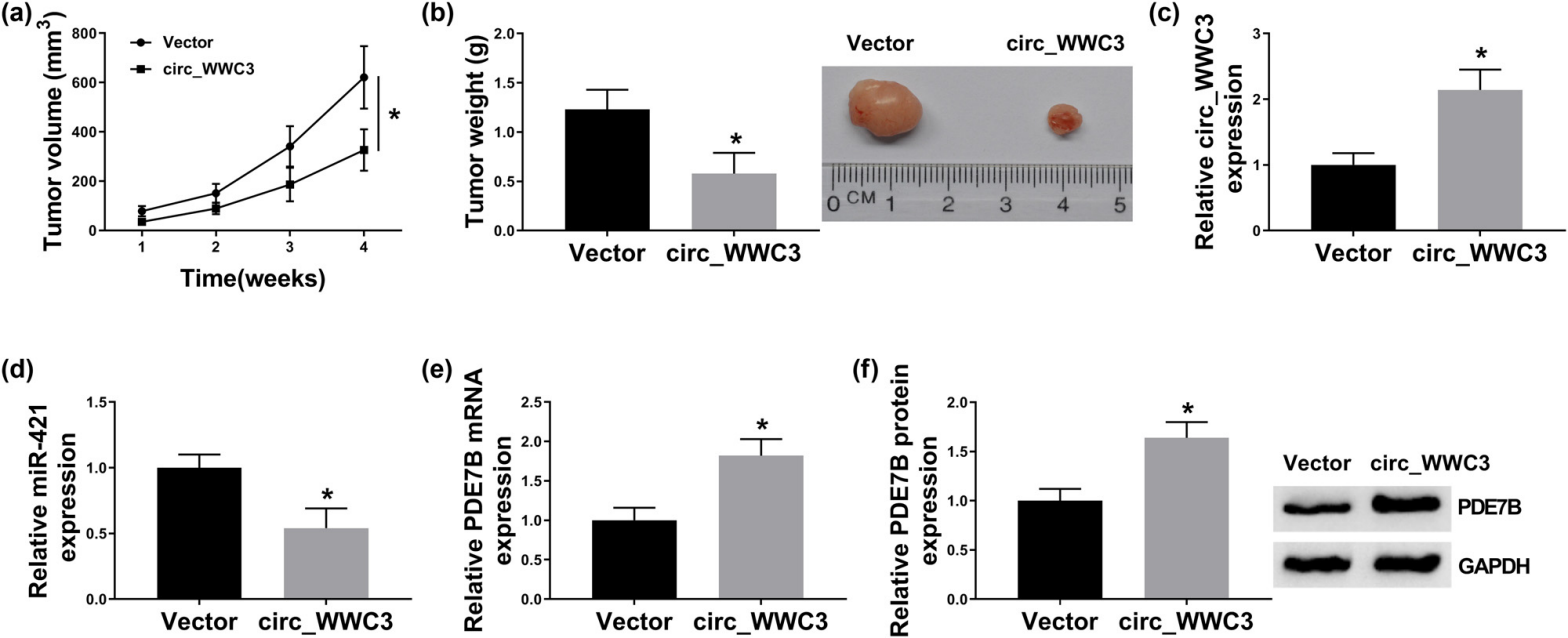
Overexpression of circ_WWC3 hampered tumor growth *in vivo*. Circ_WWC3 or Vector transfected MG63 cells were injected into the mice. (a) Tumor volume was monitored every week. (b) Tumor weight was measured after 4 weeks. (c–e) The levels of circ_WWC3, miR-421, and PDE7B in tumors collected from the mice treated with circ_WWC3 or vector were measured by qRT-PCR assay. (f) The protein level of PDE7B in tumors collected from the mice treated with circ_WWC3 or vector was measured by western blot assay. *P < 0.05.

To determine the tumorigenic function of circ_WWC3 *in vivo*, MG63 cells overexpressing circ_WWC3 were inoculated into the nude mice. After the inoculation, tumor volume was examined every week and tumor weight was measured after 4 weeks. As exhibited in Figure 7a and b, mice treated with circ_WWC3 showed decreased tumor volume and weight compared to control mice. Moreover, the treatment with circ_WWC3 elevated the levels of circ_WWC3, PDE7B mRNA, and PDE7B protein and reduced the level of miR-421 (Figure 7c–f). Collectively, circ_WWC3 overexpression restrained tumorigenesis of OS *in vivo*.

## 4 Discussion

Currently, mounting evidence has reported the vital roles of circRNAs in the carcinogenesis of cancers, including OS. As a member of circRNAs, the functions of circ_WWC3 in OS have not been clarified yet. Thus, in this research, we focused on the effects of circ_WWC3 on OS development. Our results identified that circ_WWC3 overexpression decelerated the tumorigenicity of OS by decreasing miR-421 and increasing PDE7B.

CircRNAs can function as ceRNAs to suppress miRNAs activity and further modulate the downstream gene expression [22]. Some circRNAs have been confirmed to play a tumor-suppressive role in OS. For example, circ_0002052 was abnormally decreased in OS, and the overexpression of circ_00002052 promoted OS cell apoptosis and repressed cell proliferation and motility by modulating miR-1205/APC2 axis [23]. Circ-ITCH level was demonstrated to be reduced in OS, and its elevation impeded OS cell viability and metastasis and accelerated apoptosis through interacting with miR-222 [24]. Low level of circ_0001105 was associated with poor prognosis of OS patients and circ_0001105 overexpression repressed tumor growth by interacting with miR-646 and modulating YTHDF2 expression [25]. The reports indicated that circRNAs could function as a tumor suppressor in OS. Nevertheless, there has been no report of circ_WWC3 in OS. Our research, for the first time, identified the effects of circ_WWC3 on the malignant phenotypes of OS. We verified that circ_WWC3 was aberrantly declined in OS tissue specimens and cell lines. Functionally, the elevation of circ_WWC3 restrained the colony formation, proliferation, migration, and invasion of OS cells. Moreover, circ_WWC3 upregulation facilitated OS cell apoptosis, concomitant with the downregulation of antiapoptotic protein Bcl-2 and the upregulation of pro-apoptotic proteins Bax and cleaved caspase-3. Additionally, murine xenograft model assay demonstrated that circ_WWC3 elevation blocked the tumorigenesis of OS *in vivo*. The above results illustrated that low level of circ_WWC3 was responsible for the tumorigenicity of OS.

Subsequently, we further investigated the underlying molecular mechanisms of circ_WWC3 in regulating OS development. We affirmed that circ_WWC3 served as a sponge for miR-421 to enhance PDE7B expression. QRT-PCR test showed that miR-421 level was elevated and PDE7B level was reduced in OS. Loss-of-function experiments indicated that inhibition of miR-421 impeded OS cell growth and metastasis and facilitated apoptosis. Moreover, miR-421 overexpression restored the impacts on the malignant characteristics of OS cells mediated by circ_WWC3, indicating that circ_WWC3 modulated OS cell progression via acting as the sponge for miR-421. In support of our findings, Liang et al. claimed that miR-421 level was enhanced in OS, and miR-421 suppression blocked OS cell proliferation and metastasis and promoted apoptosis by targeting LTBP2 [16]. Ren et al. disclosed that miR-421 level was linked to poor survival and worse clinical characteristics of OS patients, and miR-421 overexpression aggravated malignant biological behaviors of OS cells by interacting with MCPIP1 [17]. Though miR-421 has been identified to target multiple mRNAs to alter tumor progression, such as FOXO4 [15], MEF2D [26], and MTA1 [27], whether miR-421 can target PDE7B remains to be uncovered. Herein, we found that PDE7B could be targeted by miR-421 in OS cells. Moreover, we explored the effect of PDE7B on OS cell progression for the first time. Our results suggested that PDE7B elevation suppressed OS cell growth and motility and induced apoptosis. Deficiency of PDE7B partially rescued the impact of miR-421 suppression on OS cell progression, indicating that miR-421 contributed to OS progression by binding to PDE7B.

Taken together, our results verified that circ_WWC3 exerted the tumor-suppressive function in OS through repressing proliferation and metastasis and promoting apoptosis, possibly by modulating miR-421/PDE7B axis. Our findings expanded our understanding on the pathogenesis of OS and might provide a novel avenue for OS therapy.
